# Lights, location, action: shade avoidance signalling over spatial scales

**DOI:** 10.1093/jxb/erae217

**Published:** 2024-05-20

**Authors:** Pierre Gautrat, Sanne E A Matton, Lisa Oskam, Siddhant S Shetty, Kyra J van der Velde, Ronald Pierik

**Affiliations:** Laboratory of Molecular Biology, Wageningen University and Research, Wageningen, The Netherlands; Laboratory of Molecular Biology, Wageningen University and Research, Wageningen, The Netherlands; Laboratory of Molecular Biology, Wageningen University and Research, Wageningen, The Netherlands; Experimental and Computational Plant Development, Institute of Environmental Biology, Utrecht University, Utrecht, The Netherlands; Laboratory of Molecular Biology, Wageningen University and Research, Wageningen, The Netherlands; Laboratory of Molecular Biology, Wageningen University and Research, Wageningen, The Netherlands; Experimental and Computational Plant Development, Institute of Environmental Biology, Utrecht University, Utrecht, The Netherlands; Laboratory of Molecular Biology, Wageningen University and Research, Wageningen, The Netherlands; Indian Institute of Science Education and Research, India

**Keywords:** Cryptochrome, light signalling, photobiology, phototropin, phytochrome, shade, shade avoidance, signalling

## Abstract

Plants growing in dense vegetation need to flexibly position their photosynthetic organs to ensure optimal light capture in a competitive environment. They do so through a suite of developmental responses referred to as the shade avoidance syndrome. Below ground, root development is also adjusted in response to above-ground neighbour proximity. Canopies are dynamic and complex environments with heterogeneous light cues in the far-red, red, blue, and UV spectrum, which can be perceived by photoreceptors in spatially separated plant tissues. Molecular regulation of plant architecture adjustment via PHYTOCHROME-INTERACTING FACTOR transcription factors and growth-related hormones such as auxin, gibberellic acid, brassinosteroids, and abscisic acid were historically studied without much attention to spatial or tissue-specific context. Recent developments and technologies have, however, sparked strong interest in spatially explicit understanding of shade avoidance regulation. Other environmental factors such as temperature and nutrient availability interact with the molecular shade avoidance regulation network, often depending on the spatial location of the signals, and the responding organs. Here, we review recent advances in how plants respond to heterogeneous light cues and integrate these with other environmental signals.

## Teaser

This review summarizes recent advances in plant light signalling and associated developmental responses in the context of shade avoidance, with an emphasis on spatially explicit regulation.

## Introduction

Plants typically grow in dense stands, often monocultures in agricultural fields and diverse mixtures of species in natural fields. Both above and below ground, this leads to strong interactions between individuals within the community. Although some of these interactions can be neutral or even positive (Novoplansky, 2009; Huber *et al.*, 2021), they mostly involve competitive interactions for resource acquisition. Below ground, resource competition typically relates to mineral nutrients and/or water, whereas above ground competition is for light. Since resources are not equally distributed around the plant, responses need to be spatially coordinated. At the same time, some of the signalling involved to optimize nutrient acquisition and usage is influenced by systemic information from other plant parts (De Kroon *et al.*, 2009).

Developmental plasticity is the key property of plants to adjust their architecture such that it optimally facilitates resource capture. An important example of such plasticity is the shade avoidance syndrome (SAS): a suite of responses that collectively improve leaf positioning for light capture in dense vegetation. Well-established SAS traits are accelerated stem elongation, upward leaf movement (hyponasty), and apical dominance, together helping to position the photosynthetically active leaves in the higher, light-exposed canopy layers (reviewed in Fernández-Milmanda and Ballaré, 2021; Casal and Fankhauser, 2023). In addition, chloroplasts within the leaf cells also optimize their intracellular distribution to maximize light harvesting, or avoid excess light, further contributing to optimized light harvesting (Wada, 2013). Phototropism, asymmetric shoot growth towards relatively high blue light intensity, is another growth response through which a seedling is able to reposition its shoot (Liscum *et al.*, 2014). Ultimately, flowering can be accelerated to ensure some reproductive output under highly competitive conditions. Most of our understanding of the molecular pathways regulating these developmental adjustments is based on controlled-environment experiments with homogeneic light treatments. Plants grow in highly heterogeneic and dynamic canopies, where light signals are positioned specifically in space and time.

To enable shade avoidance responses to light signals of neighbour proximity, plants need to prioritize where to invest their resources. The long-standing idea of passive resource competition within the organism has been replaced by the understanding that molecular mechanisms are in place to prioritize one response over the other. This prioritization during SAS includes suppression of investments into root development (van Gelderen *et al*., 2018) and defence responses against attackers (reviewed in Pierik and Ballaré, 2021), but also prioritizing carbon allocation towards elongating petioles at the expense of leaf blades (de Wit *et al*., 2018). At the same time, such networks can also act to modulate shade avoidance when stresses such as soil salinity or nutrient deficiencies occur (Hayes *et al*., 2019; van Gelderen *et al*., 2021). Thus, both neighbour proximity signals and developmental responses are spatially defined over the plant body and interact in the context of other environmental signalling routes that operate simultaneously.

Here we review recent insights and advances into understanding how plants combine spatially heterogeneic light signals and molecular pathways that operate across spatial scales in the plant body to optimize plant architecture for light competition.

## Canopy light signals and perception

### Neighbour proximity and canopy shade signals

**Fig. 1.**
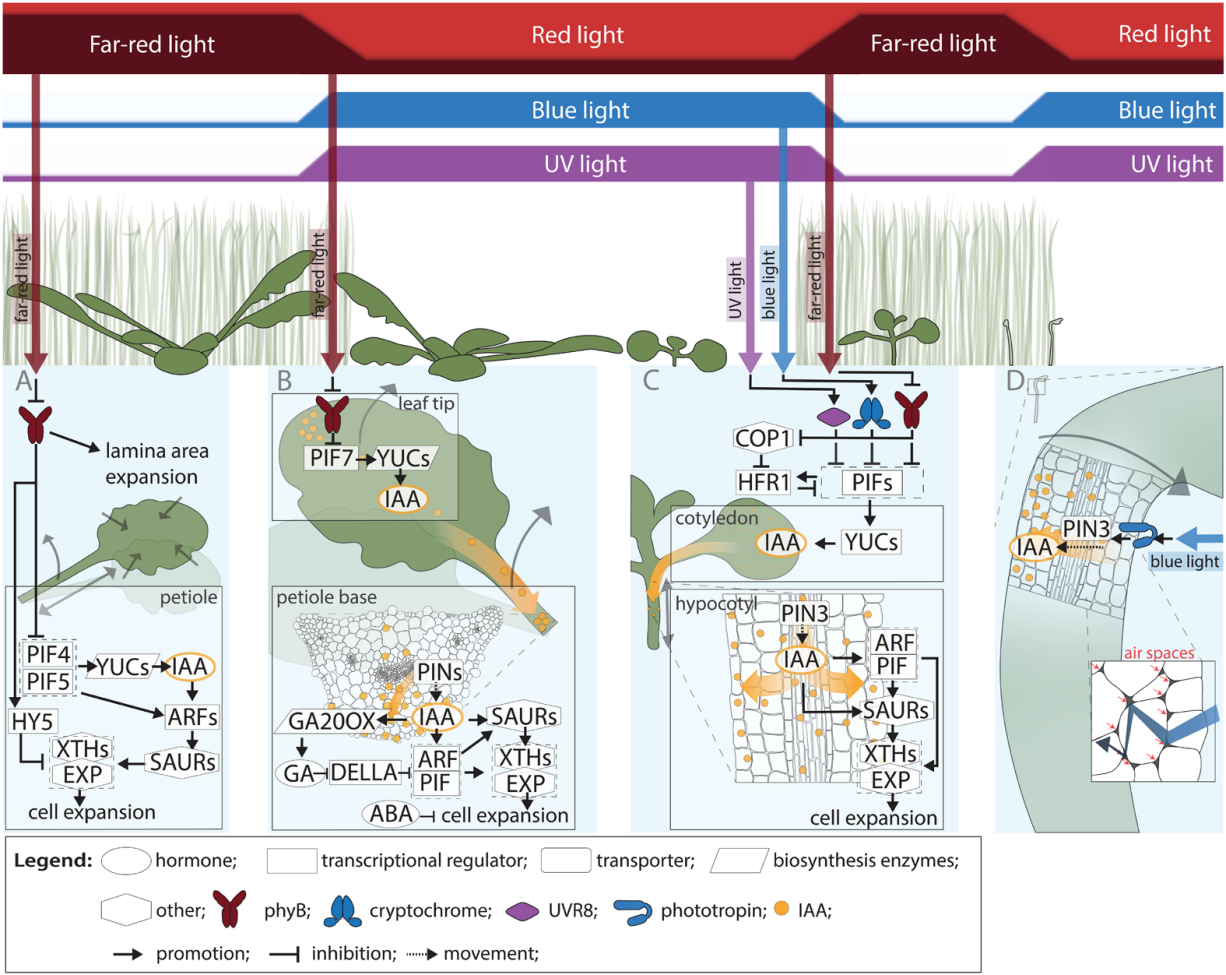
Above ground responses to low R:FR, blue and UV light. Different plant responses are elicited by different light signals and their combinations, which are associated with varying light quality conditions inside, outside, and adjacent to a canopy. The three top bars represent the change in R:FR ranging from high to low, blue- and UV-light intensity. Molecular pathways associated with each phenotype are represented in the lower panels (A–D). In all pathways depicted, auxin (IAA, actors and intermediates represented in orange) plays an important role in inducing turgor dependent cell expansion. (A) Lamina size reduction and petiole elongation. Adult Arabidopsis grown in low R:FR displays phyB dependent lamina size reduction and petiole elongation. (B) Hyponasty. Adult Arabidopsis leaves exposed to local low R:FR on the leaf tip display phyB-dependent hyponasty. The location of light perception and auxin biosynthesis (leaf tip) and location of differential growth (petiole base) are on opposite ends of the proximal–distal leaf axis. (C) Hypocotyl elongation. In Arabidopsis seedlings, auxin is produced upon low R:FR perception in the cotyledons and transported to the hypocotyl where it is transported from the vasculature to the epidermis. Low R:FR-induced hypocotyl elongation is attenuated by blue and UV-light. (D) Phototropism. Differential hypocotyl growth during phototropism in etiolated Arabidopsis seedlings is dependent on opposing gradients of phot activity and auxin concentration. The gradient of phot activity arises when blue light is scattered within the hypocotyl via air channels. In the bottom panel, air channels are indicated by red arrows, and diffracted blue light with the blue arrow. ABA, abscisic acid, ARF, AUXIN RESPONSE FACTOR; COP1, CONSTITUTIVELY PHOTOMORPHOGENIC 1; EXP, EXPANSIN; GA, gibberellic acid; GA20OX, GA20 OXIDASE; HFR1, LONG HYPOCOTYL IN FAR-RED1; HY5, ELONGATED HYPOCOTYL 5; IAA, indole-3-acetic acid; phyB, PHYTOCHROME B; PIF, PHYTOCHROME INTERACTING FACTOR; PIN, PIN-FORMED; SAUR, SMALL AUXIN UPREGULATED RNAs; UVR8, UV RESISTANCE LOCUS 8; XTH, XYLOGLUCAN ENDOTRANSGLUCOSYLASE/HYDROLASE; YUC, YUCCA.

In growing vegetation, the first light signal to emerge is a reduction in the red: far-red light ratio (R:FR; see Box 1 for definitions and explanations of light spectra components and experimental treatments). This results from horizontal far-red (FR) reflection by the vertical structure of neighbouring plants, thus enriching others with FR. The reflected light is not only FR rich, but also progressively red (R)-depleted because of absorption for photosynthesis. Together these lead to a FR-shift of the R:FR (Ballaré *et al.*, 1987, 1990). Historically, internode elongation responses to low R:FR were thought to occur in response to local R:FR sensing, but also via sensing in the closest leaf (Ballaré *et al.*, 1990; Casal and Smith, 1988a, b). In vegetation that has little vertical structure, for example that composed of rosette species such as Arabidopsis, FR enrichment is not the only cue for early neighbour detection. In Arabidopsis monocultures, early neighbour detection involves signalling of mechanical touching by horizontally protruding neighbouring leaf tips (de Wit *et al*., 2012; Pantazopoulou *et al.*, 2023). Mechanistically, light- and mechanostimulation-based early neighbour detection are separate pathways (Pantazopoulou *et al.*, 2023), possibly ensuring timely neighbour proximity responses. When the canopy continues developing, leaves will start to overlap, causing actual shade from the top layers onto the lower leaves. In shade, the R:FR decreases further due to severe R absorption by photosynthetic pigments. In addition, blue light is also depleted by absorption and these two signals together, low blue and low R:FR, synergistically act to promote shade avoidance responses (de Wit *et al*., 2016b). Furthermore, naturally occurring UV-B radiation is also depleted in the canopy, and UV-B in full sunlight impairs low R:FR-induced elongation (Mazza and Ballaré, 2015; Hayes *et al*., 2019). In addition to sensing these specific wavebands, the reduction in photosynthetically active radiation (PAR) by itself can also generate secondary signals, for example from the photosynthetic machinery in the chloroplasts and energy homeostasis that can impact shade avoidance (Millenaar *et al.*, 2009). Thus, depending on the developmental stage and plant location, different combinations of light cues occur (Fig. 1).

**Box 1. Glossary**

**Light spectral definitions**

PAR: photosynthetically active radiation: µmol photons m^−2^ s^−1^ (400–700 nm waveband).

WL: white light—broad spectrum background light in the PAR waveband.

UV-B: ultraviolet B (280–315 nm waveband).

UV-A: ultraviolet A (315–400 nm waveband).

Blue light (400–500 nm waveband).

R light: red light (600–700 nm waveband).

FR light: far-red light (700–800 nm waveband).

**Light treatments:**

FR light enrichment: far-red light supplementation through an additional FR light source.

EODFR: end of day far-red, achieved through a FR pulse at the end of the day that extends into the night.

R:FR: red to far-red light ratio, used in studies where FR light is added to a white light spectrum: typically, low R:FR (WL+FR) versus high R:FR (WL).

Shade: a mimic of actual shading through foliage, achieved typically through a combination of low PAR and low R:FR.

### Photoreceptors for shade avoidance

Early neighbour detection through R:FR happens before actual shading occurs and prepares plants for upcoming light competition. Phytochromes are photoreversible R:FR sensors: R light activates phytochromes and FR light typically inactivates them. Thus, when vegetation develops and the R:FR decreases, phytochrome activity decreases because a larger fraction will photoconvert from the active into the inactive form. Since the R:FR is affected specifically by the absorption properties of leaves, it is a reliable indicator of neighbouring plants. In Arabidopsis, phytochrome (phy) A–E are all influenced by the R:FR, with phyA and phyB being most abundant. Because phyA is very light-sensitive even in low light conditions, phyA is particularly relevant and abundant during seedling germination, de-etiolation, and establishment in low light conditions (reviewed in Legris *et al*., 2019). Contrarily, phyB is more sensitive to changes in R:FR in higher light conditions and regulates shade avoidance in both seedlings and adult plants. Active, FR absorbing, phytochrome (Pfr) localizes to the nucleus where it forms membrane-less structures called photobodies (reviewed in Pardi and Nusinow, 2021). Inactive, or R absorbing phytochrome (Pr) localizes in the cytoplasm. Once the canopy closes, all wavebands become depleted, and dedicated photoreceptors detect these changes. Blue light is sensed through several photoreceptors, with cryptochrome (cry) 1, cry2, phototropin (phot) 1, and phot2 being the primary sensors involved in responses to neighbours. UV-B light is sensed principally through UV Resistance Locus 8 (UVR8), but phot1/2 are also able to detect UV-B light (Vandenbussche *et al.*, 2014). UV-A light can be sensed by phyA, phototropins, and cryptochromes, but responses to UV-A light are poorly investigated (reviewed in Verdaguer *et al*., 2017).

Cryptochromes regulate various processes, such as photomorphogenesis, shade avoidance and entrainment of the circadian clock (Wang and Lin, 2020). UV-A and -B light activates cryptochromes through conformational changes, requiring phosphorylation and photo-oligomerization (Ponnu and Hoecker, 2022). Activation of cry is fluence rate-dependent, with cry1 requiring higher fluence rates compared with cry2 (Liu *et al.*, 2020). Both cry1 and cry2 are active in the nucleus, with cry1 shuttling between the nucleus and the cytoplasm, and cry2 remaining in the nucleus. Although cry1 and cry2 have partially overlapping functions, because of their different light-sensitivities, cry1 is considered the main cryptochrome involved in attenuating shade avoidance responses (Ponnu and Hoecker, 2022).

Phototropins mostly regulate growth towards blue light, known as phototropism (reviewed in Legris and Boccaccini, 2020; Haga and Sakai, 2023). Phototropism is primarily regulated by phot1, with a minor role for phot2. On the other hand, phot2 is the main photoreceptor controlling chloroplast movement in response to light intensity, optimizing photosynthesis (reviewed in Łabuz *et al.*, 2022). Both phot1 and phot2 are localized to the plasma membrane and chloroplast membrane (Kong *et al.*, 2013). When activated by blue light, phototropins promote phosphorylation of themselves and other proteins involved in phototropism (reviewed in Legris and Boccaccini, 2020). UVR8 is a specific UV-B light photoreceptor and was found to attenuate shade avoidance responses in both hypocotyl elongation (Hayes *et al*., 2014) and leaf hyponasty (Mazza and Ballaré, 2015). UVR8 exists either as an inactive dimer or as an active monomer. Activated by UV-B, UVR8 becomes nuclear localized where it alters gene expression (reviewed in Legris and Boccaccini, 2020). UVR8 is dependent on CONSTITUTIVELY PHOTOMORPHOGENIC 1 (COP1), an E3 ligase also involved in low R:FR signalling, for nuclear localization as UVR8 itself does not contain a nuclear localization sequence (reviewed in Liang *et al*., 2019). Photoreceptor signalling pathways can act independently, but also interact to modulate the expression of shade avoidance traits, thus allowing plants to fine-tune their architecture to dynamic competition scenarios (de Wit *et al*., 2016b; Goyal *et al.*, 2016; Boccaccini *et al.*, 2020; Tissot and Ulm, 2020).

## Molecular pathways for shade avoidance

### Photoreceptor inactivation and release of PIF inhibition is essential for shade avoidance

Molecular regulation of low R:FR-induced shade avoidance starts with phytochrome inactivation (Fig. 1A–C). Active phytochromes induce phosphorylation of PHYTOCHROME INTERACTING FACTORS (PIFs) in the nucleus, a family of basic helix–loop–helix (bHLH) transcription factors playing a central role in shade avoidance (reviewed in Cheng *et al*., 2021). This phosphorylation results in either degradation of PIF1, PIF3, PIF4, and PIF5 or inactivation of PIF7 (Leivar and Monte, 2014). PIF7 stability and abundance is further maintained via Ubiquitin Specific Proteases (UBP) 12 and UBP13 (Zhou *et al*., 2021). Both PIF1 and PIF3 contain binding domains for phyA, whereas all PIFs contain binding domains for phyB (Leivar and Monte, 2014; Oh *et al.*, 2020). Interaction of phyB with PIF5 and 7 was recently shown to occur in nuclear photobodies (Kim *et al*., 2023; Zhu *et al.*, 2023). However, PIF7 inhibition has also been reported to involve cytoplasm retention in high R:FR and nuclear localization in response to low R:FR (Huang *et al.*, 2018).

Phytochrome inactivation by FR enrichment and subsequent release of PIF inhibition allows PIFs to ensure transcriptional regulation (Sellaro *et al.*, 2017). Different PIFs have shared and separate target genes and regulate different parts of SAS either in conjunction or separately (Sellaro *et al.*, 2017). The SAS contributions of PIF1, PIF3, PIF4, and PIF5 have often been studied together using the quadruple mutant *pifq* in seedlings (reviewed in Pham *et al*., 2018). Loss of their function resulted in a more pronounced reduction in neighbour evasion responses than observed in lower order mutants (Leivar *et al.*, 2012). PIF4, PIF5, and PIF7, however, are considered the main PIFs involved in the induction of hypocotyl and petiole elongation, and hyponasty (reviewed in Pierik and Ballaré, 2021). Although these three PIFs are all important for low R:FR responses, only PIF4 and PIF5 are necessary for hypocotyl elongation responses to blue light depletion, which is regulated via cryptochromes (Pedmale *et al.*, 2016). The hyponastic response to low R:FR in adult plants is primarily induced by PIF7, with additional roles for PIF4 and PIF5 (Pantazopoulou *et al.*, 2017; Küpers *et al.*, 2023).

Besides activating downstream targets that enhance shade avoidance, several PIFs also activate their own repressors by enhancing transcription of additional non-DNA-binding bHLH transcription factors, which function as a feedback loop to curtail negative side effects of over-elongation (reviewed in Buti *et al*., 2020). PIF3-LIKE 1 (PIL1), LONG HYPOCOTYL IN FAR-RED1 (HFR1), and PHYTOCHROME RAPIDLY REGULATED 1 (PAR1) and PAR2 are all well-characterized examples that interact with PIFs, inhibiting PIFs from binding to their target gene promoters. These PIF-inhibiting HLH proteins are in turn degraded by the E3 ligase COP1, which forms a complex with SUPPRESSOR OF PHYA-105 (SPA) proteins. COP1 function is regulated by phytochromes (Sheerin *et al.*, 2015) and is active in low R:FR (Pacín *et al.*, 2013; Costigliolo Rojas *et al.*, 2022), repressed by UVR8 in high UV-B (Tavridou *et al.*, 2020), and repressed by cry1 and cry2 in high blue light (Ponnu *et al.*, 2019). Other PIF-induced negative regulators of PIF activity are the HECATE proteins HEC1 and HEC2. HECs, reminiscent of PAR and HFR1, are also HLH proteins that physically interact with PIF1, thereby preventing its DNA binding and transcriptional activation ability (Zhu *et al.*, 2016). Although these proteins were shown to affect photomorphogenesis, it is unknown if they also affect shade avoidance.

Moreover, PIFs can also enhance phyA and phyB expression (Zhu *et al.*, 2023). This feedback loop has additional layers of complexity in the form of PIF7-linked activation of the long non-coding RNA (lncRNA) *PUAR*, which blocks the binding of PIF7 to the *PHYA* promoter, hindering activation of transcription (Zhu *et al.*, 2023). Suppressing the *PUAR* transcript reduced the hypocotyl elongation response in low light and low R:FR. These extensive layers of regulation allow fine-tuned control of gene expression of downstream targets, and thus phenotypic responses to canopy shade nuances.

### PIFs regulate auxin homeostasis

PIFs elicit shade avoidance responses either directly via activation of downstream targets that are growth- or elongation-promoting, or indirectly via amplifying auxin biosynthesis through activation of *YUCCA* (*YUC*) genes *YUC2*, *YUC5*, *YUC8*, and *YUC9* (reviewed in de Wit *et al*., 2016a) (Fig. 1A–C). YUC enzymes convert indole-pyruvic acid to indole-3-acetic acid (IAA, auxin), the rate-limiting step in tryptophane-derived auxin biosynthesis (Mashiguchi *et al.*, 2011; Stepanova *et al.*, 2011; Won *et al.*, 2011). TEOSINTE BRANCHED 1, CYCLOIDEA, and PCF (TCP) transcription factors can co-regulate shade avoidance responses in Arabidopsis in a phytochrome-independent route, and the triple *tcp5 tcp13 tcp17* mutant has a reduced hypocotyl elongation response to low R:FR (Zhou *et al*., 2018). TCP17 activates expression of *PIF4* and *PIF5*, as well as the downstream targets *YUC2*, *YUC5*, and *YUC8* independently of PIFs (Zhou *et al*., 2018).

Most of the shade avoidance responses involve organs that are not themselves the main site of auxin production. IAA transport is, therefore, crucial to deliver information about the light environment to the site of response. PIN-FORMED (PIN)-dependent polar IAA transport in low R:FR mainly involves PIN3, PIN4, and PIN7 (Keuskamp *et al.*, 2010; Kohnen *et al.*, 2016; Michaud *et al.*, 2017; Pantazopoulou *et al.*, 2017; Küpers *et al.*, 2023). Lateral IAA transport via PINs in the hypocotyl is essential for blue light-induced phototropism, and is dependent on phot1 (Ding *et al.*, 2011) (Fig. 1D). Additionally, there will also be some auxin diffusion from cell to cell via plasmodesmata (Gao *et al.*, 2020). The triple *pin3 pin4 pin7* mutant displays severely reduced elongation and hyponasty in low R:FR (Michaud *et al.*, 2017; Pantazopoulou *et al.*, 2017; Küpers *et al.*, 2023). PIN kinases PINOID (PID) and WAG2 influence PIN polar membrane localization, and their gene expression is influenced by lncRNA *AUXIN-REGULATED PROMOTER LOOP* (*APOLO*) (Mammarella *et al.*, 2023). Low R:FR inhibits *APOLO* expression, and mutants with altered *APOLO* expression levels showed a reduced hyponastic response in low R:FR.

Auxin/indole acetic acid (AUX/IAA) proteins are degraded when auxin mediates the binding between auxin receptor complex components Transport Inhibitor Resistant 1 (TIR1), Auxin Signalling F-Box (AFB) and AUX/IAAs, releasing the AUXIN RESPONSE FACTOR (ARF) transcription factors (reviewed in Leyser, 2018). Active cry1 and phyB can stabilize AUX/IAAs under blue and R light conditions (Xu *et al.*, 2018). Once released from their inhibition when auxin is present, ARFs can regulate expression of their target genes. PIFs interact with several ARFs, of which ARF6, ARF7, and ARF8 are jointly involved in low R:FR responses (Reed *et al.*, 2018; Küpers *et al.*, 2023).

### Auxin regulates cell wall modifications

Cell wall modifications are key to allow turgor-driven cell expansion, and different groups of cell wall modifying proteins are involved in shade avoidance (Sasidharan *et al*., 2008, 2010) (Fig. 1A–C). These proteins are activated by acidification of the apoplast, following plasma membrane H^+^-ATPase activity (reviewed in Du *et al*., 2020). *SMALL AUXIN UPREGULATED RNAs* (*SAURs*) are rapidly induced when auxin is present (reviewed in Stortenbeker and Bemer, 2019) and SAUR19 can activate plasma membrane H^+^-ATPases (Spartz *et al.*, 2014). ARFs have been implicated in activating SAUR transcription, as TIR1 and auxin are required for rapid plasma membrane H^+^-ATPase phosphorylation (Uchida *et al.*, 2018). PIF7 and TCP13 can also bind directly to the promoter region of *SAUR19–23* and PIF7 does so under low R:FR conditions (Willige *et al.*, 2021; Hur *et al.*, 2024). Cell wall modifying proteins such as EXPANSIN and XYLOGLUCAN ENDOTRANSGLUCOSYLASE/HYDROLASE (XTH) enable cell wall loosening (reviewed in Du *et al.*, 2020) and their activities are regulated during, and required for, shade avoidance (Sasidharan *et al*., 2008, 2010). Finally, XTHs are inhibited by ELONGATED HYPOCOTYL 5 (HY5) and HY5 HOMOLOGUE (HYH), two negative regulators of low R:FR induced shade avoidance responses (Sharma *et al.*, 2023).

### Gibberellins, brassinosteroids, and abscisic acid regulate shade avoidance

Besides auxin, other hormones are involved as well in molecular regulation of low R:FR responses. Gibberellic acid (GA) biosynthesis genes, especially *GA20 OXIDASES*, are up-regulated during low R:FR exposure (Hisamatsu *et al.*, 2005; Küpers *et al.*, 2023), which in turn enhances the degradation of DELLA proteins in low R:FR (Djakovic‐Petrovic *et al.*, 2007). DELLAs can physically interact with and inhibit activity of PIFs (de Lucas *et al.*, 2008; Feng *et al.*, 2008), and additionally inhibit other positive growth regulators such as ARFs and brassinosteroid signalling actors BRASSINAZOLE RESISTANT 1 (BZR1) and homolog BRI1-EMS-SUPPRESSOR 1 (BES1) (Oh *et al.*, 2014; Li *et al*., 2016) (Fig. 1B). The BZR1–ARF6–PIF4/DELLA (BAP/D) transcription factor module has been proposed to integrate these hormone signalling routes and enable organ specific responses depending on the cellular and spatial context (reviewed in Bouré *et al.*, 2019). Abscisic acid (ABA) has been consistently implicated in regulation of low R:FR responses as ABA biosynthesis is increased during low R:FR (Küpers *et al.*, 2023; Michaud *et al.*, 2023). ABA is involved in branching suppression during low R:FR, together with PIF4 and PIF5 (Holalu *et al.*, 2020), with low R:FR enhancing endogenous ABA levels via the PIF module (Michaud *et al.*, 2023). At the same time, ABA supresses most branching-unrelated shade avoidance responses, and indeed a *nced3 nced5* ABA biosynthesis double mutant is constitutively hyponastic in high R:FR conditions already (Michaud *et al.*, 2023).

### Temporal changes in light intensity during sunflecks are sensed by photoreceptors

Sunflecks are short temporal peaks in light intensity in the understory of a canopy. These variations range in the order of seconds to minutes and are caused by wind-induced movement of the foliage (reviewed in Smith and Berry, 2013). These short peaks of increased light intensity add additional complexity to canopy low R:FR signals for plants, because of their brief nature and sudden shifts in irradiance. Seedlings grown in low R:FR and exposed to white light + UV light sunflecks show less hypocotyl elongation compared with seedlings exposed to just low R:FR, and sunflecks are detected by phyB, cry1, cry2, and UVR8 (Belmonte *et al.*, 2024, Preprint). UVR8 dimer/monomer ratio is influenced by sunflecks, with a higher dimer fraction in low UV-B conditions, and a shift towards monomers, the active conformation, in sunflecks or higher UV-B light conditions (Moriconi *et al.*, 2018). Both UVR8 and cry1 abundance in the nucleus are increased during sunflecks (Belmonte *et al.*, 2024, Preprint). The rate at which phyB can transition between active and inactive state is a limiting factor that ultimately affects the accuracy at which phyB activity follows R:FR fluctuations in between sunflecks (Sellaro *et al.*, 2019).

## Spatial heterogeneity of signals and responses: above ground

### Organ specific low R:FR responses in seedlings

The above-described molecular interactions from supplemental FR-induced phyB inactivation to cell elongation occur at spatially distinct sites of the organism (Fig. 1C). In *Brassica rapa* seedlings, auxin synthesis is enhanced in the cotyledons upon low R:FR-exposure, and IAA is subsequently transported to the hypocotyl where it promotes epidermal cell elongation (Procko *et al*., 2014). In Arabidopsis, PIF7 promotes this cotyledon-based auxin synthesis by promoting *YUC* gene expression, facilitating *de novo* IAA synthesis (Das *et al.*, 2016; Kohnen *et al.*, 2016). *YUC* expression is mainly observed in the mesophyll and epidermis of the cotyledon, compared with the vasculature upon end of day FR (EODFR) exposure (Kim *et al*., 2018). Auxin response genes, on the other hand, were mostly up-regulated in the vasculature. Once cotyledon-derived IAA has reached the hypocotyl, it is transported from the vasculature towards the epidermis (Keuskamp *et al.*, 2010), where it drives hypocotyl elongation (Procko *et al*., 2016). The auxin translocation depends on PIN3, PIN4, and PIN7 auxin efflux carriers (Keuskamp *et al.*, 2010; Kohnen *et al.*, 2016). In addition to auxin transport from the cotyledons controlling hypocotyl IAA levels, these can also be modified directly in the hypocotyl by (de)-conjugation of IAA via GH3.17, independent of IAA synthesis (Zheng *et al.*, 2016). The auxin response is further facilitated in the hypocotyl by HISTONE DEACETYLASE 9 (HDA9), which co-induces several SAUR and AUX/IAA proteins under low R:FR light conditions (Nguyen *et al.*, 2023). It is thought that PIF4 and PIF5 are especially important for auxin responsiveness, whereas PIF7 seems particularly important for promoting auxin synthesis (Li *et al*., 2012; Hersch *et al.*, 2014; Pantazopoulou *et al.*, 2017).

Seedlings can integrate multiple shade cues, notably low R:FR and blue light intensity for hypocotyl elongation and phototropic bending (de Wit *et al*., 2016b; Goyal *et al.*, 2016). It is mostly unknown if these signals are integrated within the same spatial location. This would be expected if their integration involves early photoreceptor signalling interactions (de Wit *et al*., 2016b), but interactions can also occur further downstream in the signalling cascades that take place in different locations. A recent study shows how low blue light promotes autophagy in both hypocotyls and cotyledons, likely facilitating recycling of resources for stem elongation under resource-limiting conditions (Ince *et al.*, 2022), providing no evidence for tissue-specificity in this specific element of shade avoidance regulation.

### Spatial tissue configuration for sensing light direction in phototropism

Even though the phototropin-based detection of light direction and the subsequent formation of an auxin gradient across the diameter of the hypocotyl have been well described (reviewed in Legris and Boccaccini, 2020), the question how there can be a light gradient over the diameter of an etiolated hypocotyl was only recently resolved. It was found that intercellular air spaces between cortex cells in the Arabidopsis hypocotyl cause light diffraction, leading to a gradient of blue light between individual cells in the hypocotyl when light is only coming from one direction (Nawkar *et al.*, 2023). This gradient of blue light facilitates a gradient of phot1 activation, subsequently leading to a PIN-dependent auxin gradient required to induce differential growth (Fig. 1D). The role of air spaces in phototropic bending towards other wavelengths, such as UV-B is not yet known. Also in Arabidopsis leaves and *Brachypodium distachyon* coleoptiles, water infiltration and thereby filling up of air spaces increased the light transmission, but associated phenotypic responses are not yet studied (Nawkar *et al.*, 2023). Phototropins have a prominent role not only in seedling phototropism but also in leaves, where they regulate, for example, leaf flattening (Legris *et al*., 2021). A normal *PHOT1* expression pattern between abaxial and adaxial sides of the leaf is needed for adequate leaf development (Legris *et al*., 2021). It is possible that light gradients created by air spaces present in leaves also function in a developmental process like leaf flattening.

### Local low R:FR perception in leaves

Leaves of adult Arabidopsis can position themselves in a canopy through regulation of cell growth in petioles and laminas, allowing either uniform elongation or organ bending when growth rates are different between two sides, for example abaxial versus adaxial. Light control of bending and elongation of petiole and lamina result in 3D control of leaf positioning (Oskam *et al.*, 2024). Low R:FR exposure typically promotes petiole elongation through cell elongation, while often inhibiting lamina growth by limiting cell division in young leaves and limiting cell expansion in older leaves (Romanowski *et al.*, 2021; Hussain *et al.*, 2022) (Fig. 1A). Low R:FR at the leaf tip induces petiole hyponasty via differential cell elongation on the basal abaxial section of the petiole without additional petiole elongation, unlike whole plant exposure to low R:FR, which induces both petiole elongation and hyponasty (Michaud *et al.*, 2017; Pantazopoulou *et al.*, 2017; Küpers *et al.*, 2023) (Fig. 1B). Low R:FR exposure at the leaf tip enhances *YUC2*, *YUC5*, *YUC8*, and *YUC9* expression in the leaf tip specifically (Küpers *et al.*, 2023). Which cell layers in low R:FR are important in adult plants for light perception or auxin biosynthesis is currently unknown. The auxin synthesized through this pathway in the leaves is transported to the petiole base, where an auxin gradient between the abaxial and adaxial side is set up throughout all cell layers (Küpers *et al.*, 2023). PIN3 localization showed a predisposition for the abaxial side of petiole bundle sheath cells already in control light, which became more pronounced upon auxin enrichment from the leaf tip (Küpers *et al.*, 2023). An increase in abaxial PIN3 localization was also observed during hyponastic leaf movement under elevated temperature (Park *et al.*, 2019). In low R:FR-exposed *pin3 pin4 pin7* mutants, auxin accumulation on the abaxial petiole side was reduced and restricted to the cell layer directly surrounding the vasculature (Küpers *et al.*, 2023). Interestingly, ABA also affects leaf angles, and it was shown recently that loss of ABA signalling through *abi1-1* expression, in the mesophyll, but not in stomata or bundle sheath, stimulates leaf angles even in the absence of additional FR light (Michaud *et al.*, 2023).

The described spatial separation of the perception and response at organ, tissue, and cellular level allows plants to fine-tune their reaction to shade to optimize leaf positioning and in a heterogenic light environment. Light signalling effects are not limited to leaf positioning, as several shoot derived factors are mobile and travel through the vasculature towards the roots.

## Root developmental responses to light

### Light influences root development

Light, through both direct and indirect routes, can shape root system architecture, hence affecting nutrient acquisition (Yeh *et al.*, 2020). This regulation is both light quality and quantity dependent, with the developmental response being influenced by the upstream perception of the light cue and the downstream signalling pathways (Ha *et al.*, 2018; Y. Yang *et al*., 2020; Gao *et al.*, 2021; Miotto *et al.*, 2021). Roots can respond to light cues by root-localized sensing of light, by the action of shoot to root endogenous mobile signals produced in response to specific light cues, or by a combination of both (Suzuki *et al.*, 2011; Ha *et al.*, 2018; van Gelderen *et al*., 2018). Root-autonomous light sensing can occur in scenarios where roots are grown in light-exposed conditions (Mo *et al.*, 2015) or when light is axially conducted primarily through the plant vasculature, from the shoots to the roots, referred to as stem-piped light (Lee *et al.*, 2016; Y. Yang *et al*., 2020).

### Direct light sensing by roots

Light can penetrate several centimetres into soil, hence directly influencing root development, although its extinction is strong. Photoreceptors are expressed in different regions of the root and have distinct roles in influencing root development in response to light exposure (Mo *et al.*, 2015). Both phytochromes (*PHYA*, *PHYB*, and *PHYD*) and *UVR8* are primarily expressed in the meristematic zone and root cap, while *PHOT1* is expressed in the upper layers of the mature root. Stem-piped light can be sensed in the root by phyB (Lee *et al.*, 2016), as well as by UVR8 (Y. Yang *et al*., 2020) receptors. Longer wavelength photons are conducted further than those of shorter wavelengths, but there is a strong attenuation in the light conduction within centimetres from the source (Sun *et al.*, 2005).

### Molecular mechanisms regulating direct sensing of light in roots

Exposing roots directly to white light as opposed to growing them in darkness represses both primary root growth and lateral root emergence. Blue light signalling mediated by cryptochromes and phototropins represses both primary and lateral root growth and is the proposed dominant pathway for light-induced inhibition of root growth under white light (Silva-Navas *et al.*, 2015). Additionally, phot1 sensing of blue light positively affects directional root growth (Muthert *et al.*, 2020).

**Fig. 2.**
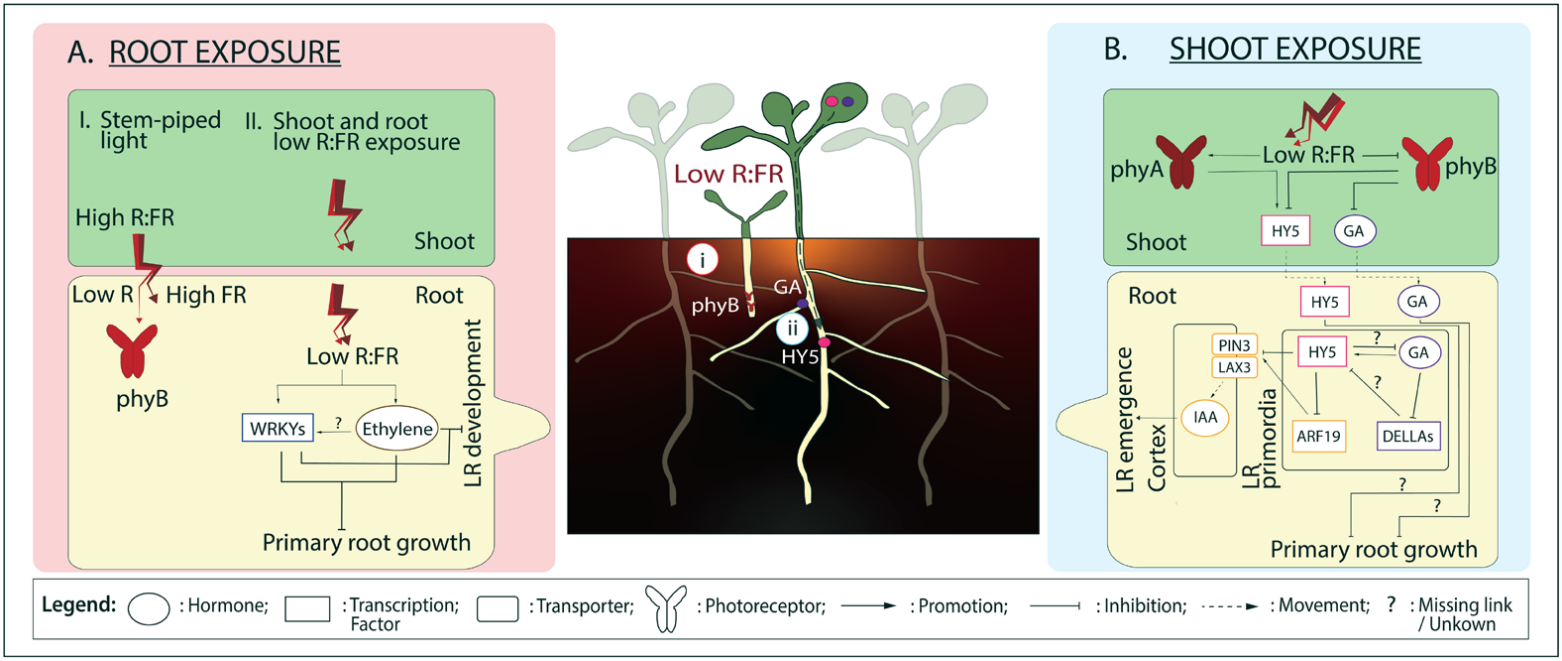
Low R:FR influences root growth and development. Low R:FR can trigger distinct molecular pathways based on the site(s) of perception. The central panel represents plants grown at high density where the light is FR-enriched. (A) In scenario (i), represented here in a younger seedling, roots are exposed to low R:FR either through stem-piped FR light or through exposure of both shoot and roots to light (transmitted through soil cracks). (B) In scenario (ii), represented here in an older seedling, light is perceived by the shoot and the signal is transmitted by mobile factors such as HY5 and GA. Actors involved in hormonal pathways and independent transcription factor families are assigned specific colours: WRKYs in dark blue, ethylene in brown, HY5 in pink, gibberellin-associated actors in purple and auxin-associated actors in orange. ARF19, AUXIN RESPONSE FACTOR 19; GA, gibberellic acid; HY5, ELONGATED HYPOCOTYL 5; IAA, indole-3-acetic acid; LAX3, LIKE AUX1 3; LR, lateral root; phyA, PHYTOCHROME A; phyB, PHYTOCHROME B; PIN3, PIN-FORMED 3; R:FR, red to far-red light ratio.

Stem piped UV-B light is sensed in the roots by UVR8, which interacts with the R2R3 type MYB domain containing transcription factors MYB73 and MYB77, inhibiting lateral root length through the repression of auxin responsive genes (Y. Yang *et al*., 2020). In addition, UV-B light can affect root growth via the DUF647 domain containing proteins, ROOT UV-B SENSITIVE1/2 (RUS1 and 2), and the loss of function *rus1* mutants display severely stunted shoot and root development upon root exposure to light (Tong *et al.*, 2008; Leasure *et al.*, 2009). Stem piped light can induce the nuclear localization of phyB in roots (Lee *et al.*, 2016). Additionally, combined exposure of shoot and roots to low R:FR can result in inhibition of lateral and primary root growth in Arabidopsis seedlings (Rosado *et al.*, 2022). This response is mediated by WRKY transcription factors and ethylene signalling. WRKY26 and WRKY45 inhibit lateral root emergence and primary root growth, while WRKY75 inhibits only lateral root emergence. Ethylene is proposed to act upstream of the WRKYs in the low R:FR root response (Rosado *et al.*, 2022) (Fig. 2). It is unknown if the WRKY-mediated response to FR exposure and phyB-mediated sensing of stem-piped light are parts of the same root-autonomous light sensing and response pathway, or if the WRKY route acts in shoot-to-root signalling.

The plant’s response to photoperception by the roots can partially represent a stress reaction that is accompanied by a burst of reactive oxygen species (Muthert *et al.*, 2020). In the root apical meristem, this photoperception is followed by a HY5-mediated reduction of H_2_O_2_ levels. This reduction happens both directly via the transcriptional activation of the PER6 peroxidase, and indirectly via the repression of the transcriptional repressor of peroxidase genes by the transcription factor UPBEAT1. Both processes aid in the alleviation of the inhibition of primary root growth (Li *et al*., 2024).

Direct exposure of roots to white light can also result in shoot phyB-induced abscisic acid signalling via ABA INSENSITIVE 5 (ABI5), which can transcriptionally activate the peroxidase PER1, reducing H_2_O_2_ levels and hence promoting root growth (Ha *et al.*, 2018). Both these mechanisms are employed by the plant to alleviate the reactive oxygen species-mediated inhibition of root growth in response to direct exposure to light.

### Shoot to root signalling is required to shape root system architecture in dense canopies

While roots can directly perceive light with photoreceptors as described above, roots do typically grow in darkness deeper in the soil and therefore depend on light perception by the shoot. Plants that compete for light in dense canopies give priority to shade avoidance responses to light depletion and low R:FR in order to capture sufficient light for photosynthesis, while suppressing root development in this process (van Gelderen *et al*., 2018). Reduction of light intensity for shoots specifically reduces primary and lateral root development with sucrose and auxin proposed as possible long-distance signals in this response (Kircher and Schopfer, 2012, 2023; Miotto *et al.*, 2021). Exogenous sucrose promotes primary root growth and lateral root formation, and signalling via photosynthetic glucose is required for root meristem activation (Xiong *et al.*, 2013; Miotto *et al.*, 2021). Photosynthetic sugars are produced in the leaves, and since photosynthesis can be compromised at high planting densities, sugars are possible candidates for shoot-to-root signalling in low light conditions (Bailey *et al.*, 2001; Miotto *et al.*, 2021). Glucose affects auxin metabolism, transport, and signalling and this could explain how sugars can affect root development in low light conditions, apart from their important function as a resource (Mishra *et al.*, 2009; Miotto *et al.*, 2021). However, several reports show that low R:FR conditions can also promote photosynthesis in crops such as soybean and lettuce (Zhen and van Iersel, 2017; F. Yang *et al*., 2020). Therefore, sugar availability might be increased in low R:FR conditions, as shown in Arabidopsis (Yang *et al*., 2016) and tomato (Courbier *et al*., 2020), arguing against sugar reductions as the cause of root phenotypes in these conditions. In response to low R:FR, primary root growth and lateral root formation are reduced (Salisbury *et al.*, 2007; van Gelderen *et al*., 2018; Rosado *et al.*, 2022). As mentioned before, directly exposing roots to light can already affect root development (Cabrera *et al.*, 2022) and it is therefore desired to use growth systems in which roots are kept in darkness (Silva-Navas *et al.*, 2015; Bontpart *et al.*, 2020). Dark root systems facilitate research on root phenotypes that depend on shoot-perceived light cues such as low R:FR and the associated mobile regulators. Indeed, the root phenotype in low R:FR is apparent when only shoots are illuminated with low R:FR and roots are kept in the dark (van Gelderen *et al*., 2018).

Since shoot auxin synthesis is elevated in low R:FR and lateral root density is still affected by low R:FR in higher order *yuc* mutants, auxin, through reduced delivery to the root, is not likely the mobile factor that reduces root development in response to low R:FR (van Gelderen *et al*., 2018). HY5 can move from the shoot to the root via the phloem and is degraded after ubiquitination by the COP1–SPA complex (Huang *et al.*, 2014; Chen *et al.*, 2016). In low R:FR (Zheng *et al.*, 2013) phyA is activated in seedlings and is proposed to indirectly stimulate HY5 levels because of its negative effect on COP1 (Osterlund *et al.*, 2000; Rausenberger *et al.*, 2011; van Gelderen *et al*., 2018). HY5 represses lateral root formation, and HY5 protein levels are elevated in lateral root primordia (LRPs) of seedlings of which the shoots are in low R:FR (van Gelderen *et al*., 2018). HY5 was shown to decrease PIN3 and LIKE AUX1 3 (LAX3) plasma membrane abundance in the cortex cells overlying the LRPs of seedlings in low R:FR (van Gelderen *et al*., 2018) (Fig. 2).

In addition to HY5, other factors like the hormone GA can move from shoot to root. GA promotes root growth when present in optimal concentrations in the roots (Shani *et al.*, 2013; Regnault *et al.*, 2015; Tal *et al.*, 2016; Barker *et al.*, 2021). GA is needed to maintain and promote root meristem size and GA located in the endodermis of the root elongation zone regulates the cell expansion in this zone (Ubeda-Tomás *et al*., 2008, 2009). Bioactive levels of GA are increased by low R:FR in the shoot, and the resulting reduction of DELLA-mediated inhibition on PIFs promotes the shade avoidance responses (reviewed in Fernández-Milmanda and Ballaré, 2021). The root response to supplemental FR detected by the shoot appears to be affected by shoot-to-root transport of GA and this may interact with HY5 (van Gelderen *et al*., 2023, Preprint) (Fig. 2). GA accumulates in LRPs in response to shoot-perceived low R:FR and exogenous GA treatment on the shoot leads to a reduction of root development (van Gelderen *et al*., 2023, Preprint).

### Low R:FR perceived by the shoot inhibits lateral root primordia emerging from the main root

Lateral roots can be formed throughout most of the life of the plant and therefore lateral root development is influenced by many different environmental cues, including a reduction in lateral root number by low R:FR perception in the shoots. Lateral roots are initiated in the xylem pole pericycle, starting with the division of lateral root founder cells. LRP development is characterized by further division of these cells, forming a dome-shaped LRP that eventually emerges from the main root. The development of the LRP is divided into seven stages, from the first division in the pericycle (stage 1) up to the moment the LRP is about to emerge from the main root (stage 7) (Malamy and Benfey, 1997). Low R:FR perceived by the shoot can lead to a stalling in stages 1 + 2 and especially 5 + 6 LRPs, resulting in fewer stage 7 LRPs that emerge (van Gelderen *et al*., 2018) (Fig. 2). During development, the LRP passes three cell layers (endodermis, cortex, and epidermis) to eventually emerge from the main root (reviewed in Vilches-Barro and Maizel, 2015). These layers need to give way to the developing LRP to become a visible lateral root. Auxin perception is required in the endodermis cells overlaying the primordium to let the LRP pass this cell layer by drastically changing shape of the overlaying cells (Swarup *et al.*, 2008; Lucas *et al.*, 2013; Vermeer *et al.*, 2014). The LRP crosses the endodermis after stage 2, indicating that this cell layer likely limits early LRP development in low R:FR conditions, where some LRPs are stalled in stage 1 + 2 (Vilches-Barro and Maizel, 2015; van Gelderen *et al*., 2018). The next layers, cortex and epidermis, are crossed by pushing cells apart via an auxin-dependent induction of cell wall remodelling enzymes in cells overlaying the LRP, and these cells hardly change shape during this process (Swarup *et al.*, 2008; Kumpf *et al.*, 2013). Auxin derived from the LRP induces a signalling cascade involving ARF7 and ARF19 in the cortex and epidermis, inducing expression of PIN3 and LAX3, which causes a further accumulation of auxin in these cells (Swarup *et al.*, 2008; Péret *et al.*, 2013). In low R:FR conditions, it was shown that plasma membrane abundance of LAX3 and PIN3 on the cortex cells overlaying the LRP was decreased compared with high R:FR conditions (van Gelderen *et al*., 2018) (Fig. 2). This explains the increase of stage 5 + 6 LRPs, because the impaired auxin accumulation in the overlaying cortex cells prevents the LRP from emerging from the main root (van Gelderen *et al*., 2018).

While substantial progress has been made in understanding the effect of canopy-derived FR enrichment on root development, several open questions remain. These include understanding the integration of FR enrichment responses with other cues affecting root development, such as foraging responses for mineral nutrients. Comprehension of the tissue/cell specificity of such regulations is also a contemporary challenge. Recent technological advances that allow monitoring of root growth in soil may also help in increasing the temporal resolution of these root responses to FR enrichment in non-invasive experimental set-ups (Pree *et al.*, 2024, Preprint).

## Environmental factors impacting shade avoidance responses

Plants growing in natural or agricultural set-ups are never subjected to a single environmental variation but always to a myriad of cues, perceived by both roots and shoots, that need integration to ultimately adopt the most adequate developmental responses for survival and successful reproduction. Three integration scenarios are possible if we consider two environmental cues named *A* and *B*: *A* can affect the response to *B*, or vice versa, or both *A* and *B* can influence each other’s responses. Shade avoidance signalling and responses are no exception, and their integration with other environmental cues is a rising field of research. FR light enrichment can affect the responses to other environmental cues; this is typically the case with the attenuation of defence against pathogens (Chico *et al.*, 2014; Fernández-Milmanda *et al.*, 2020) or wounding responses (Fiorucci *et al.*, 2022). Such modulation of biotic, or abiotic stress responses by light quality has been thoroughly reviewed by Courbier and Pierik (2019) and Fernández-Milmanda and Ballaré (2021). Here, we will focus on how environmental cues can affect shade avoidance responses and involve spatially explicit signals and responses.

### Synergistic effects of warmth and FR light enrichment on shoot development

**Fig. 3.**
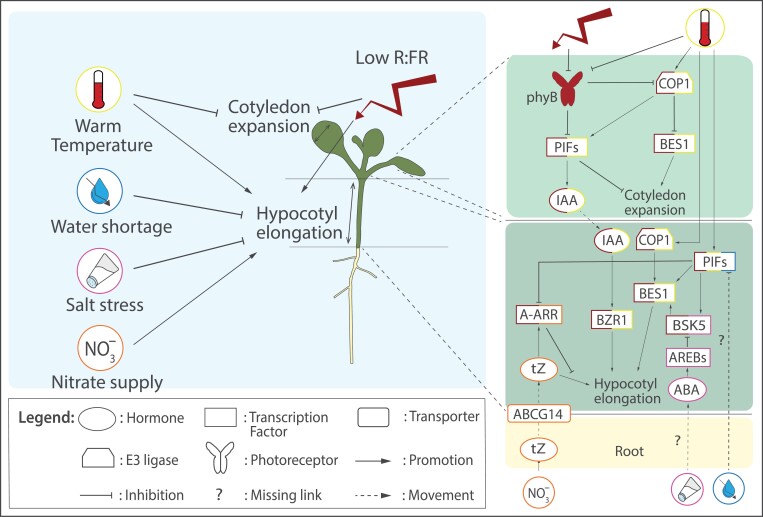
Environmental factors impacting shade avoidance responses and underlying molecular actors. Top left panel represents the overall phenotypic outcome of warm temperature, water shortage, salt stress, and nitrate supply on shade avoidance responses, at seedling stage, with promotion of hypocotyl elongation and reduction of cotyledon expansion as phenotypes of interest. The right panels from top to bottom represent the cotyledons, the hypocotyl, and the roots. Each environmental cue and associated molecular actors are represented by a specific colour: low R:FR in red, warm temperature in yellow, water shortage in blue, salt stress in pink, and nitrate supply in orange. Molecular actors outlined with multiple colours are integrators, meaning that they are regulated by multiple environmental cues. For example, COP1, outlined in red and yellow, is regulated by both low R:FR and warm temperatures. Root regulations and phenotypes associated with the environmental cues have been omitted. A-ARR, Type A ARABIDOPSIS RESPONSE REGULATOR; ABA, abscisic acid; ABCG14, ATP-binding cassette (ABC) transporter subfamily G14; AREB, ABA-RESPONSIVE ELEMENT-BINDING FACTOR; BES1, BRI1–EMS–SUPPRESSOR 1; BRZ1, BRASSINAZOLE RESISTANT 1; BSK5, BRASSINOSTEROID SIGNALING KINASE 5; COP1, CONSTITUTIVELY PHOTOMORPHOGENIC 1; IAA, indole-3-acetic acid; phyB, PHYTOCHROME B; PIF, PHYTOCHROME INTERACTING FACTOR; R:FR, red to far-red light ratio; tZ, *trans*-zeatin.

A notable example of shade avoidance response integration with other environmental cues is its convergence and synergistic effect with warm temperatures. Warm temperatures (up to ~30 °C for Arabidopsis) lead to a series of developmental responses collectively known as thermomorphogenesis (reviewed in Casal and Balasubramanian, 2019). While elevated temperature and low R:FR have different effects on root architecture (van Gelderen *et al*., 2018; Casal and Balasubramanian, 2019), the effects of elevated temperature on shoot architecture are strikingly similar to shade avoidance responses: hypocotyl/petiole/stem elongation, leaf hyponasty, reduction of leaf area, and early flowering. Such similarities between those two environmental stimuli have been explained by a convergence towards the same molecular actors. Indeed, phyB is not only a photoreceptor, but also a thermosensor: warmer temperatures promote conversion of active phyB Pfr into inactive Pr (Jung *et al.*, 2016; Legris *et al*., 2016). This leads to the de-repression of the pathways involved in shade avoidance, eliciting similar responses. Together with this phyB regulation, PIF4, PIF5, and PIF7 are also further activated by warmth independently of phyB, through transcriptional, translational, or post-translational mechanisms, which have been reviewed by Casal and Fankhauser (2023). Low R:FR and warmth-triggered hypocotyl elongation both require synthesis of auxin (downstream of the phyB/PIF module) in the cotyledons and its translocation to the hypocotyl, and this promotes brassinosteroid signalling with the BZR1 transcription factor playing a major role, leading to cell elongation (Procko *et al*., 2014; Bellstaedt *et al.*, 2019). Both low R:FR and warmth also influence accumulation of the growth promoting transcription factor BES1 (Costigliolo Rojas *et al.*, 2022). Strikingly, COP1 represses BES1 accumulation in the cotyledons, yet promotes it in the hypocotyl (Fig. 3). This highlights how similar actors can have an organ-specific wiring, thus leading to opposite developmental responses: a reduction of cotyledon growth and a promotion of hypocotyl growth in response to FR light enrichment and warmth. Interestingly, simultaneous low R:FR and warmth promotes elongation more than the additive effect of the individual cues (Burko *et al.*, 2022). This synergy requires PIF7 and auxin transport from cotyledon to hypocotyl but other molecular components, yet unknown, are also necessary to understand the mechanisms involved as PIF7 activity and auxin responses are similar in FR-enriched conditions, independently of the temperature.

### Root-borne abiotic stresses negatively impact shade avoidance responses

While environmental factors perceived by the shoot, such as temperature, can impact shade avoidance responses, some root stresses also strongly affect them. For example, very mild soil salinity levels can already reduce low R:FR-induced elongation responses (Hayes *et al*., 2019). This salt-triggered regulation depends on the canonical ABA signalling pathway, seems to occur mostly through the osmotic effect of salt, and leads to a reduction of the transcriptional induction by low R:FR of *BRASSINOSTEROID SIGNALING KINASE 5* (*BSK5*), which is necessary for the activation of BES1. Intriguingly, while salt leads to an overall inhibition of low R:FR-induced elongation, the kinase essential for salt tolerance, SALT OVERLY SENSITIVE2 (SOS2), was found to directly phosphorylate PIF4 and PIF5 in a way that reduces interaction with phyB, thus promoting their accumulation and elongation during combined shade and salt stress (Han *et al.*, 2023). This highlights how a cue can have an overall negative impact on shade avoidance responses, while also subtly modulating them for fine tuning. Water restriction can also reduce FR-induced hypocotyl elongation through a transcriptional down-regulation of *PIF3*, *4*, and *5*, while the reduction of cotyledon area remains unaffected (Semmoloni *et al.*, 2022, Preprint). Osmotic stress thus inhibits shade avoidance responses, but not the growth of all organs, hinting towards localized molecular responses depending on the organ context (Fig. 3).

While progress on understanding the molecular mechanisms of salt- and drought-mediated attenuation of shade responses has been made, a key piece of information is missing: what are the mobile actors from root to shoot signalling the stresses? Established signalling intermediates for osmotic stress might have a wider range of action than what was already described, and perhaps impact shade responses. Two prime candidates are ABA and the CLAVATA3/EMBRYO-SURROUNDING REGION-RELATED 25 (CLE25) peptide. While root ABA production was shown not to be required for stomatal closure in response to dehydration (Christmann *et al.*, 2007), ABA translocation from root to shoot by the ABCG25 transporter can regulate stomata opening under non-stressed conditions (Yang *et al*., 2024). It is therefore not to be excluded that root-borne ABA impacts shade avoidance responses, especially since ABA reduces hypocotyl elongation in response to FR enrichment (Hayes *et al*., 2019) and regulates leaf angles (Michaud *et al.*, 2023). The CLE25 peptide is produced under osmotic stress, is root-to-shoot mobile, and can cause stomatal closure through stimulation of ABA production in the shoot (Takahashi *et al.*, 2018). It would be interesting to know if CLE25 would be able to locally induce ABA synthesis to reach levels capable of modulating shade avoidance responses in low R:FR light conditions.

### Nutrient availability shapes shade avoidance responses

In dense vegetation stands, while there is competition above ground for light harvesting, the same is also true underground, where plants must compete for nutrients that are often distributed heterogeneously and in limiting amounts. Limited knowledge is available on how nutrient availability impacts shade responses and vice versa. While the impacts of R light through phyB, PIFs and HY5, and low R:FR through jasmonic acid on inorganic phosphorus (P_i_) uptake have been well described (Sakuraba *et al.*, 2018; Sun *et al.*, 2023), the impact of P_i_ availability on shade avoidance responses has been barely studied. Indeed, low P_i_ availability seems to decrease low R:FR-induced hypocotyl elongation and abolishes the low R:FR-induced reduction of lateral root densities (van Gelderen *et al*., 2021), but the molecular mechanisms involved are unknown.

Recent studies have given more insight into the integration of shade avoidance responses and nitrogen nutrition. Although nitrate nutrition affects shade avoidance responses, it appears that other sources of nitrogen such as ammonia or amino acids do not (van Gelderen *et al*., 2021; Pereyra *et al.*, 2023). Compared with sufficient or high nitrate concentrations, nitrate depletion strongly reduces low R:FR-induced hypocotyl elongation (van Gelderen *et al*., 2021), mostly through mechanisms involving root-derived cytokinins (Gautrat *et al.*, 2023, Preprint). In response to nitrate, a specific species of cytokinin, *trans*-zeatin (tZ), is produced in roots and then translocated to shoots via the ABCG14 transporter (Ko *et al.*, 2014; Zhang *et al.*, 2014; Sakakibara, 2021). tZ was found to positively regulate hypocotyl elongation only under low R:FR conditions. This specificity of action was explained by sensitization of hypocotyls to tZ in low R:FR conditions thanks to a down-regulation of type-A *ARABIDOPSIS RESPONSE REGULATORS* (*ARRs*), which are negative regulators of cytokinin signalling. The down-regulation of type-A *ARRs* is dependent on FR-mediated phyB inactivation and PIFs, mainly PIF7 (Gautrat *et al*., 2023, Preprint; Fig. 3). Intriguingly, while low R:FR promotes hypocotyl elongation, it also inhibits cotyledon growth, and both seem to be coordinated through cytokinins. In the cotyledons specifically, up-regulation of the cytokinin degrading enzyme *CYTOKININ OXYDASE 5* (*CKX5*) expression leads to a lowering of active cytokinin in the cotyledons, leading to reduced growth (Carabelli *et al.*, 2007), whilst sensitization to tZ in the hypocotyl allows local, cytokinin-dependent growth promotion. This allows the fine-tuning of shoot morphology in response to supplemental FR light by integrating nitrate-derived cues. Interestingly, a shift from low to high nitrate in standard control light conditions promotes hypocotyl elongation, and this can occur shoot-autonomously, thus not relying on shoot-to-root mobile information (Pereyra *et al.*, 2023). Although classic nitrate homeostasis factors, such as the NIN-LIKE PROTEIN 7 (NLP7) transcription factor and the NRT1.1 transporter are involved in this response, also factors that are strongly associated with shade avoidance and thermomorphogenesis, such as PIF4, auxin, and *SAUR* genes are required (Pereyra *et al.*, 2023). In conclusion, there is a wide array of signalling interactions that can modulate shade responses in tissue-specific manners, allowing for intricate and organ-specific optimizations based on a multidimensional environmental information.

## Conclusion and future perspectives

Navigating in a canopy towards the best lit patches requires developmental plasticity of shoot organs in response to heterogeneous light cues. Recent advances have opened insights into spatial and cellular context of shade avoidance regulation. Many mobile components, including hormones, are required to bring information to specific sites of cellular responses, as in the case of leaf movements and root developmental plasticity. Recent technological advances, such as single cell sequencing, hold the promise to spatially resolve regulation of information processing over organ, tissue, and even cellular scales.

It has also become apparent that shade avoidance is not just regulated through R:FR signalling but involves integration of multiple light cues. If and how this integration of information happens in the same tissues or cells is largely an open question. Furthermore, the molecular mechanisms through which these light signalling pathways are integrated with endogenous signalling routes triggered by other environmental cues are only starting to be investigated. These interactions of pathways may also involve spatially distinct plant compartments. Once such interactions are thoroughly understood, translating this knowledge to crop plants holds promise for optimizing plant developmental plasticity to planting densities and thus further fine-tuning allocation to yield components.
